# Determination of Monacolin K and Citrinin in the Presence of Other Active Ingredients Found in Selected Food Supplements by HPLC-DAD

**DOI:** 10.3390/molecules31010016

**Published:** 2025-12-20

**Authors:** Urszula Hubicka, Barbara Żuromska-Witek, Marek Szlósarczyk, Ewelina Sołtys, Martyna Rusak, Izabela Gacal

**Affiliations:** Department of Inorganic Chemistry and Pharmaceutical Analytics, Faculty of Pharmacy, Jagiellonian University Medical College, 9 Medyczna Street, 30-688 Kraków, Poland; barbara.zuromska@uj.edu.pl (B.Ż.-W.); m.szlosarczyk@uj.edu.pl (M.S.);

**Keywords:** monacolin K, lovastatin, citrinin, determination, HPLC, food supplements, quality control

## Abstract

A universal HPLC method with diode array detection was developed for the separation and determination of the lactone and acid forms of monacolin K in the presence of other active ingredients (vitamins B_1_, B_6_, B_12_, and folic acid) found in selected dietary supplements. The method also enables the quantitative determination of citrinin in monacolin K. Chromatographic separation was performed on an ACE 5 C18-PFP column (250 × 4.6 mm) thermostated at 25 °C. The mobile phase consisted of 0.005 M phosphate buffer (pH 2.60) and acetonitrile under linear gradient elution conditions. Detection was carried out spectrophotometrically at 230 nm for monacolin K and 325 nm for citrinin. The total run time was 28 min. The method was validated and met the acceptance criteria for specificity, linearity, sensitivity, accuracy, and precision. Linearity was achieved over a broad concentration range: 12.48–37.44 μg·mL^−1^ for MK and 3.48–5.22 μg·mL^−1^ for CTN. The method is sufficiently sensitive, with LOD and LOQ values of 0.91–2.85 μg·mL^−1^ and 2.18–3.48 μg·mL^−1^ for MK and CTN, respectively. Good precision (RSD < 0.70%) and intermediate precision (RSD < 1.33%) were observed. The accuracy of the method, expressed as percentage recovery at three concentration levels, ranged from 98.73% to 100.64%. The analysis revealed that the monacolin K content in randomly selected dietary supplements did not comply with the manufacturer’s declaration in any case.

## 1. Introduction

Monacolin K (MK), a natural statin, inhibits HMG-CoA reductase (3-hydroxy-3-methylglutaryl-CoA) reversibly, blocking the conversion of HMG-CoA to mevalonate, a key step in cholesterol synthesis [1]. MK is produced during rice fermentation with yeast strains *Monascus* spp., as the secondary metabolite [2]. Traditionally used in East Asian cuisine for its colour, flavour, and preservative properties, fermented red rice is now popular for managing hyperlipidemia and is sold in dried or powdered forms [1].

**Figure 1:**
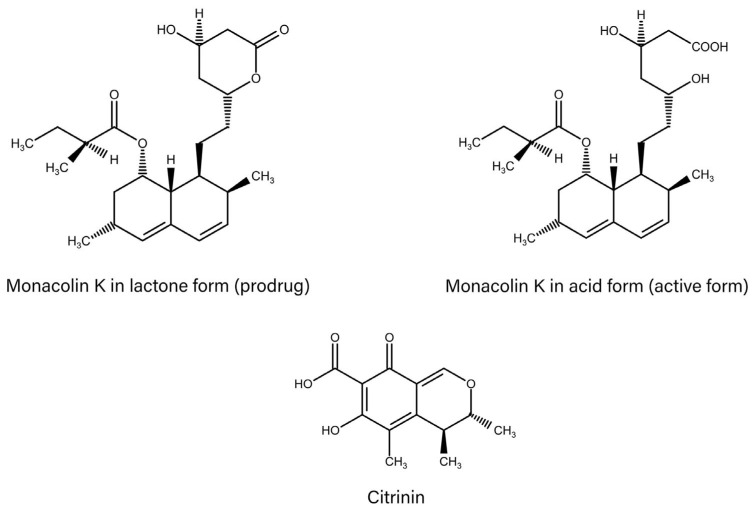
Chemical structures of lactone and acidic forms of Monacolin K and citrinin.

MK was isolated from red yeast rice (RYR) for the first time in 1975 in Japan [3]. For the fermentation process, *Monascus purpureus* fungi and other organisms, including *Aspergillus terreus* and *Penicillium* spp., are the most commonly used [4]. During fermentation, a range of secondary metabolites is generated, including pigments, monacolins (predominantly MK), as well as polysaccharides, γ-aminobutyric acid (GABA), ergosterol, and citrinin [1].MK can exist in two forms (lactone and acid) (Figure 1), which, at the appropriate pH, interconvert into each other [5,6]. The synthetic drug lovastatin (approved in 1987 by the FDA as the first statin drug) and MK in lactone form (MK-L) have an identical chemical structure and show a similar hypolipemic effect in in vivo tests [7]. MK-L and lovastatin undergo hydrolysis in the liver to the hydroxy acid form, which represents the pharmacologically active form [5]. Lovastatin is recognised as a well-documented synthetic drug; meanwhile, MK is an element of the RYR product [4,6,8].

Lovastatin and MK lower total cholesterol, LDL, and triglycerides, while raising HDL. They also exhibit pleiotropic effects, including plaque stabilisation, antioxidant activity, and improved vascular endothelial function [9]. Scientific studies suggest that these substances may inhibit the deposition of amyloid plaques in neurons of the central nervous system, potentially delaying Alzheimer’s progression and lowering mortality risk [10]. Red yeast rice extracts, containing hypolipemic compounds, starch, sterols, and isoflavones, have shown preventive effects against obesity [11] and diabetes [12]. Moreover, recent scientific studies on MK suggest that it may also exert anticancer, anti-inflammatory and hepatoprotective effects [3].

The average prescribed dose of lovastatin ranges from 10 to 80 mg per day [1], whereas recommended doses for RYR-based products marketed as dietary supplements containing MK have varied over time [13]. In 2011, the MK dose was established at 10 mg per day, as approved by the European Food Safety Authority (EFSA) [9,14]. In 2022, a European Commission regulation reduced this level to less than 3 mg per day [9,14]. Moreover, some popular products have been prohibited and temporarily disappeared from the market [15]. The use of MK-containing preparations is limited by potential adverse effects, including headaches, gastrointestinal symptoms, myalgias, skin rashes, and severe complications such as renal or hepatic dysfunction and rhabdomyolysis [13].

Besides MK itself, another issue is the toxic substance citrinin (CTN), which can form during red rice fermentation and is a common contaminant in RYR products, with levels often higher than those of other mycotoxins [16]. This mycotoxin is hepatotoxic and nephrotoxic, and possibly carcinogenic (Group 3 by the International Agency for Research on Cancer). The mechanism of CTN toxic action is not fully understood. Its toxicity and genotoxicity may involve oxidative stress and increased mitochondrial membrane permeability, with additional evidence of embryotoxic and immunotoxic effects [17,18]. Currently, the level of CTN in preparations is regulated (Commission Regulation (EU) 2019/1901), and its maximum content in dietary supplements can be 0.1 ppm (100 µg/kg) [19].

Analytical procedures for MK determination are based on advanced chromatographic and spectroscopic techniques. The most commonly reported method in the literature is reversed-phase high-performance liquid chromatography with photodiode array detection (RP-HPLC-DAD) using a C18 column, which enables the simultaneous quantification of both the lactone and acid forms of MK and is characterised by good selectivity and repeatability [20,21,22,23]. To increase sensitivity and allow accurate structural identification, liquid chromatography coupled with mass spectrometry (LC-MS), including high-resolution techniques such as LC-QToF-MS, is widely employed, as it enables comprehensive analysis of multiple monacolins in the complex matrices of dietary supplements [24,25]. An additional method used for the quantitative assessment of major monacolins is ^1^H-NMR spectroscopy, which offers high measurement reproducibility and does not require individual analytical standards [25]. A recent publication also presented an ultrahigh-performance liquid chromatography (UHPLC) method with a DAD detector for the quantification of both the lactone and acid forms of MK in extract from RYR, using a C4 column (Kromasil, 100, 50 × 3 mm, 2.5 µm) with a mobile phase consisting of ACN and a 0.1% acetic acid solution in water (50:50 *v*/*v*) [26].

For CTN, the available literature indicates the need for a highly sensitive analytical method due to its low levels in food. The method most frequently applied for its determination is UHPLC with fluorescence detection (UHPLC-FL), which provides very low limits of detection (1.5 μg/kg) and enables rapid analysis of large numbers of samples [27,28]. To improve selectivity and sensitivity, and to confirm analyte identity, LC-MS or LC-MS/MS is widely used and considered the most reliable tool for CTN analysis in food and food supplements [16,24,29].

One of the critical issues is the quality of commercial RYR products, which have variable MK content and lack standardisation, making it difficult to assess their safety. It should be emphasised that dietary supplements are generally not subjected to standardised or stringent quality control procedures, which may result in variability in composition and potential safety concerns. As early as 2014, Commission Regulation (EU) No. 212/2014 was published in the Official Journal of the European Union, stating that the presence of CTN in certain fermented red rice products is high when used in the amount required to achieve the claimed effect, which may result in patient exposure to nephrotoxicity [9,30]. Differences were found between the declared and actual monacolin content—only 50% of the supplements contained the amount declared on the label [31]. Moreover, many of the supplements analysed did not include information on the concentration of monacolin on the label [25]. A scientific report that included tests of 37 RYR dietary supplements (sold both online and at retail in the EU) for mycotoxins and natural statins revealed alarming findings. Citrinin was found in all monitored products, including pure RYR capsules and multi-ingredient preparations with standardised amounts of monacolin K, among the mycotoxins. Only four supplements had citrinin levels that complied with the EU limit, which was applicable until April 2020, while one product met the 100 μg/kg threshold introduced thereafter.

Moreover, virtually no samples met GMP-compliant standards for MK content applicable for the pharmaceutical industry (95–105% active ingredient content). Variable, but small additional amounts of simvastatin (0.1–7.5 μg per daily dose) were detected in 30 samples. Thus, an under-regulated and poorly controlled market of dietary supplements weakens both efficacy and increases the risk of adverse effects [8]. Significant concerns remain regarding the compliance of marketed products with manufacturers’ specifications, while the global red yeast rice market was valued at USD 565.4 million in 2024 and is expected to grow further [32].

For the reasons outlined above, and considering the role of MK as an important and widely used nutraceutical product, it appears essential to develop standardised methods suitable for assessing the quality of supplements containing this compound. Therefore, the purpose of our study was to develop and validate an HPLC-DAD method for the quantitative determination of MK in both its acid and lactone forms, in the presence of additional active components, including vitamins (B_1_, B_6_, B_12_), folic acid and phytosterols, as well as CTN, a potential contaminant, in dietary supplements. No similar method has yet been found in the literature. The usefulness of this work was assessed by validating and analysing randomly selected dietary supplements available on the Polish market. We are convinced that the developed method can be a valuable tool in quality control and supplement safety. The findings of these studies could significantly influence future regulations and aid in establishing robust quality control guidelines for products containing MK.

## 2. Materials and Methods

### 2.1. Reagents

Methanol, acetonitrile, and dipotassium phosphate (analytical grade), as well as orthophosphoric acid (85%, HPLC grade), were obtained from Merck (Darmstadt, Germany). HPLC grade water was prepared using an HLP 5 purification system (Hydrolab, Straszyn, Poland). Sodium hydroxide and hydrochloric acid were supplied by POCH (Gliwice, Poland). All reagents used were of HPLC grade with a purity of ≥99.9%.

### 2.2. Standard Solutions and Substances

The following standard substances purchased from Merck (Darmstadt, Germany) were used:

Lovastatin—cat. no. 75330-75-5, with ≥98% purity (HPLC), was used as a standard for MK-L.

Citrinin—cat. no. 518-75-2, ≥98% purity (HPLC).

Thiamine hydrochloride (vitamin B_1_)—cat. no. 67-03-8, ≥99% (HPLC) purity.

Pyridoxine hydrochloride (vitamin B_6_)—cat. no. 58-56-0, ≥99% (HPLC) purity.

Cyanocobalamin (vitamin B_12_)—cat. no. 68-19-9, ≥99% (HPLC) purity.

Folic acid—cat. no. 59-30-3, ≥99% (HPLC) purity.

Standard solutions were prepared in concentration ranges:

Citrinin—from 3.48 μg·mL^−1^ to 5.22 μg·mL^−1^ in methanol.

MK-L—from 12.5 μg·mL^−1^ to 37.5 μg·mL^−1^ in methanol.

MK-A—from 12.5 μg·mL^−1^ to 37.5 μg·mL^−1^. The solution was prepared by hydrolysis of the lactone form of MK as described by Yang and Hwang [33]. The hydrolysis efficiency of the lactone form of monacolin K to the acid form was tested [33]. The chromatograms obtained after hydrolysis did not detect MK-L, confirming the high reaction yield (Figure S1).

Standard solutions of B vitamins were prepared in methanol in concentrations: B_1_—1.0020 mg·mL^−1^, B_6_—1.0110 mg·mL^−1^, B_12_—0.5000 mg·mL^−1^. The standard solution of folic acid was prepared at a concentration of 0.5012 mg·mL^−1^ in a sodium carbonate solution. All the standards stock solutions were stored protected from light and freshly prepared immediately before analysis to ensure stability.

### 2.3. Food Supplements

The following food supplements were used:

I—film-coated tablets containing 10 mg monacolin K; 1.4 mg vitamin B_6_; 200 µg folic acid; 2.5 µg vitamin B_12_;

II—film-coated tablets containing 10 mg monacolin K; 150 mg phytosterols; 1.1 mg vitamin B_1_; 1.4 mg vitamin B_6_; 200 µg folic acid; 2.5 µg vitamin B_12_;

III—tablets containing 10 mg of monacolin K; 166 mg soy extract, including 99% phytosterols (165 mg); 1.1 mg vitamin B_1_; 1.4 mg vitamin B_6_; 200 µg folic acid; 2.5 µg vitamin B_12_;

IV—tablets containing 10 mg of monacolin K;

V—capsules containing 10 mg of monacolin K;

VI—tablets containing 10 mg of monacolin K;

VII—capsules containing 10 mg of monacolin K;

VIII—capsules containing 2.75 mg of monacolin K;

IX—capsules containing 2.9 mg of monacolin K;

X—tablets containing 10 mg of monacolin K, 1.10 mg of vitamin B_1_, 1.40 mg of vitamin B_6_, 2.5 µg of vitamin B_12_, and 200 µg of folic acid. The manufacturer recommends a dose of one-quarter of a tablet per day.

All food supplements used in the study were purchased from local pharmacies or online stores, each with an expiration date valid for at least one year after the date of purchase.

*Sample preparation for determination of MK*

The contents of each capsule or powdered tablet were quantitatively transferred to a 25 mL flask, extracted with 15 mL of methanol by mechanical shaking for 25 min, and subsequently sonicated for 5 min using low power settings and cooling the bath. The solution was made to volume with methanol, mixed and filtered through syringe filters. After methanol extraction, samples were first passed through 5.0 µm Millex™ Durapore™ hydrophilic PVDF syringe filters to remove particulate matter while minimising analyte adsorption. After dilution, final filtration was performed using 0.45 µm Millex™ PTFE syringe both were from MilliporeSigma (Merck, Darmstadt, Germany). Appropriate filtered sample aliquots were diluted with methanol in a volumetric flask to a final concentration of 0.0250 mg·mL^−1^, and a portion was transferred to a vial. The procedure was repeated for 5 capsules or tablets.

### 2.4. Instrumentation and HPLC Conditions

The liquid chromatography system (HITACHI High-Technologies Corporation, Tokyo, Japan) equipped with a solvent delivery pump, degasser, autosampler, photodiode array detector, and column oven was used. Chromatographic analysis of the tested substances was carried out on an ACE-5 C18-PFP column (250 × 4.6 mm, 5 μm particle size; Advanced Chromatography Technologies, Aberdeen, Scotland) coupled with a guard column. The column temperature was maintained at 25 °C. Separation was achieved using linear gradient elution (Table 1) with a mobile phase consisting of 0.005 M phosphate buffer (pH 2.60) and acetonitrile. The flow rate was 1.0 mL min^−1^, the injection volume was 10 μL, and the total run time was 28 min. Column equilibration between runs was performed for 5 min at starting gradient conditions. Detection was performed at 230 nm for MK and at 325 nm for CTN.

### 2.5. Method Validation

The HPLC method was validated in compliance with ICH guidelines with respect to specificity, linearity, sensitivity (LOD and LOQ), accuracy, precision, and robustness [34].

*Specificity*

Specificity of the method was assessed by comparing chromatograms of the pure standard substances (i.e., MK-A and MK-L, vitamins B1, B6, B12, folic acid, and CTN), the food supplement solutions, methanol extract of selected phytosterols (beta-sitosterol, campesterol, stigmasterol), and a blank chromatogram. In all obtained chromatograms, the retention time (t_R_), resolution factor (Rs), asymmetry factor (As), peak areas, and peak purity of the analysed substances were taken into account.

*System suitability*

System suitability was assessed by five replicate injections of standard solutions of MK-L, MK-A, and CTN. The system suitability parameters were defined for asymmetry factors, resolution factors, and the number of theoretical plates for the peaks of the tested compounds.

*Linearity*

The calibration plots for MK-L, MK-A, and CTN were constructed by analysing five separately prepared solutions covering a range of concentrations 50–150% for MK-L and MK-A and of concentration 80–120% for CTN as described in section Standard solutions and substances. Further analytical procedures were as described in the Instrumentation and HPLC conditions sections. Linearity was assessed in duplicate by measuring the relationship between peak areas and concentration (milligrams per millilitre). The slope of the regression line, the y-intercept, the standard deviation of slope and intercept, the correlation coefficient, R^2^ value, and standard error of residuals of the calibration curve were calculated using the programme Statistica v. 13.3. Then, to determine whether the residuals have a normal distribution, the Shapiro–Wilk statistical test was used. Autocorrelation in the random component of the linearity model was checked; therefore, the Durbin-Watson test was performed. Using the Bartlett test, the homogeneity of variances (heteroscedasticity) was assessed.

*Limit of detection (LOD) and limit of quantification (LOQ)*

Based on the residual standard deviation of a regression line (Se) and the slope (a) of the calibration plots and following the formula LOD = 3.3Se/a and LOQ = 10Se/a, the LOD and LOQ for MK-L and MK-A were estimated. Then the detection and quantification limits were validated by analysing solutions at the concentrations prepared at the detection and quantification limits. For CTN, the limits of detection and quantification were estimated based on the signal-to-noise ratio (S/N = 3 for LOD and S/N = 10 for LOQ) by comparing measured signals from samples with known low concentrations to those of blank samples and by establishing the minimum concentration at which CTN was detected.

Detection and quantification limits for CTN were determined experimentally based on signal-to-noise ratio (S/N = 3 for LOD and S/N = 10 for LOQ).

*Precision*

The repeatability of the method was determined by analysing six replicates of standard solutions of the tested substances from individual weighings. The study was performed using a 100% concentration level. Intermediate precision was obtained for the same concentration of freshly prepared solutions by different analysts, who performed the analysis over a period of one week. The results were expressed as the relative standard deviation (RSD).

*Accuracy*

The method’s accuracy was assessed by determining recovery percentages for MK-L, MK-A, and CTN. For this purpose, for MK-L, solutions of the III supplement, MK-L standard solutions, and model mixtures of the supplement were prepared with known amounts of the standard added at 90%, 100%, and 110% concentration levels. Similarly, solutions of the I supplement, MK-A standard solutions, and the corresponding model mixtures were prepared to assess the method’s accuracy for MK-A. To examine CTN recovery, solutions of the III supplement, CTN standard solutions, and model mixtures containing the supplement with known amounts of the standard were prepared at 80%, 100%, and 120% levels.

In accordance with ICH recommendations, three independent samples were prepared for each level. The resulting solutions were analysed chromatographically, and concentrations were calculated from peak areas using the linear regression equations obtained for study compounds in the linearity study.

*Robustness*

The robustness was evaluated by intentional minor modifications of the proposed method parameters. T The impact of small changes (±5% of the initial values) in the pH of the phosphate buffer, the column temperature (±1.0 °C), the detection wavelength (±11 nm), and the flow rate (±0.1 mL min^−1^) on the separation of the studied drugs was assessed.

*Application of the developed HPLC method for the determination of selected substances in food supplements*

The determination of MK-L and MK-A in selected food supplements was carried out by analysing the prepared supplement solutions as described in the section Food supplements according to the procedure described in section Instrumentation and HPLC conditions.

## 3. Results

Although global sales of dietary supplements are high, their production is not subject to GMP standards. This leads to variable ingredient content and contaminants. Monacolin K (MK) supplements often have incorrect dosages and can cause adverse reactions such as muscle pain, liver damage, and drug interactions. They can also be contaminated with citrinin. Variable MK content poses a risk of unpredictable effects and overdose. The lack of clear regulations means that MK is treated as a food ingredient, which limits oversight of its quality and safety. MK is often used by older adults with chronic diseases, where potentially dangerous interactions are possible, and its long-term effects remain poorly understood [9,17,18,19].

Therefore, we decided to develop a new universal HPLC-DAD method for the simultaneous determination of various forms of monacolin K in the presence of other active ingredients (vitamins B_1_, B_6_, B_12_, and folic acid) found in selected food supplements available in Poland. The first step was to optimise the conditions for separating MK in its two forms and other active substances, such as vitamins B_1_, B_6_, B_12_, and folic acid, present in the chosen food supplements commonly used to lower cholesterol levels. We examined the suitability of the ACE-5 C18 (250 × 4.60 mm, 5 µm particle size) and ACE-5 C18-PFP (250 × 4.60 mm, 5 µm particle size) columns as stationary phases. The usefulness of many mobile-phase gradient modes was also tested.

The optimal conditions for the separation and identification of all examined compounds were achieved using an ACE-5 C18-PFP column as the stationary phase. Preliminary experiments on a conventional C18 column yielded inadequate separation of vitamin B12 and folic acid; moreover, longer retention times were observed for CTN, MK-A, and MK-L.

**Figure 2:**
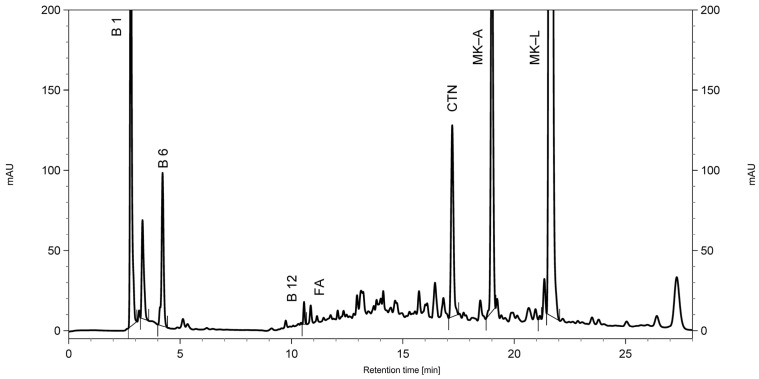
Representative chromatogram of food supplement III, spiked with citrinin standard, and recorded at 230 nm. B6—vitamin B_6_; B1—vitamin B_1_; B12—vitamin B_12_; FA—folic acid; CTN—citrinin; MK-L—lactone form of monacolin K; MK-A—acid form of monacolin K.

It was determined that the best separation of the tested compounds and good peak symmetry can be achieved using a mixture of phosphate buffer (pH = 2.60) and acetonitrile in linear gradient elution (Figure 2). The column temperature was set at 25 °C, and the flow rate was 1.0 mL min^−1^. The wavelength for spectrophotometric detection (230 nm for MK and 325 nm for CTN) was selected based on the absorption spectra of the analysed substances, recorded directly from the chromatograms.

The developed HPLC-DAD method was optimised and validated in accordance with the ICH recommendations for the validation of analytical methods [34]. The method showed that the peaks were well resolved (R > 1.5) and that the peak purity check indicated no coelution. All peaks are symmetric, with asymmetry coefficient values ranging from 1.09 to 1.35. The high theoretical plate number (N) ≥ 3000 confirms the efficiency of the chromatographic separation (Table 2).

The developed method was specific to the compounds studied. No additional peaks were observed at the methanol extract of selected phytosterols (beta-sitosterol, campesterol, stigmasterol) chromatogram (Figure S2) and the blank chromatogram, at the positions where the tested compounds occur.

A chromatographic system suitability test for MK-A, MK-L, and CTN was conducted in accordance with ICH requirements to confirm that the chromatographic system was operating correctly. Parameters such as peak area reproducibility, asymmetry coefficient, number of theoretical plates, and retention times were evaluated, and the acceptance criteria have been met (Table S1).

The regression analysis results for the examined substances are shown in Table 3.

Linearity was observed over a broad concentration range. The correlation coefficients (R) for the linear model for MK-L, MK-A, and CTN exceeded 0.99. The y-intercept values of the linear equations for MK-L, MK-A, and CTN were statistically significant. The distribution of residuals was well approximated by a normal distribution, as shown by the *p*-values (*p* > 0.05) of the Shapiro–Wilk normality test. The Durbin-Watson and Bartlett tests confirmed the absence of autocorrelation and the homogeneity of residuals in the regression models. Based on the regression analysis results, the calibration data were assumed to fit a linear model.

The method’s sensitivity was satisfactory. The LOD and LOQ values for MK-A were 0.91 μg·mL^−1^ and 2.77 μg·mL^−1^, respectively, while for MK-L, they were 0.94 μg·mL^−1^ and 2.85 μg·mL^−1^, respectively. The LOD and LOQ for CTN were 2.18 μg·mL^−1^ and 3.48 mg·L^−1^, respectively (Table 3). Good precision (RSD < 0.70%) and intermediate precision (RSD < 1.33%) were observed. The accuracy of the methods, expressed as percentage recovery at three concentration levels, ranged from 98.73% to 100.64%. Detailed results are provided in Table 4.

Under deliberately varied chromatographic conditions (flow rate, temperature of the column, and pH of the phosphate buffer in the mobile phase), the compounds tested were adequately resolved, and the elution order remained unchanged. Changes in the detection wavelength significantly affect peak area and only slightly affect the number of theoretical plates for MK-A and MK-L.

The proposed HPLC-DAD method was successfully applied to the determination of the investigated compounds in food supplements. Our research was carried out over several years. We started when the permitted dose of MK was 10 mg. Now, it has been reduced to less than 3 mg due to new regulatory requirements [31].

The selected preparations for the study were in capsule and tablet form. The results of quantitative analysis, together with statistical evaluation, are presented in Table 5.

Food supplements were analysed assuming an acceptance criterion of 95–105% of the content declared by the manufacturer for the average content of the active substance. The adopted criteria used in pharmacy are justified by high selectivity, good accuracy and repeatability of the method. Moreover, the analysed ingredients are present at relatively high concentrations, and the sample matrices are comparatively simple and do not cause interference. None of the tested preparations met the requirements. Among food supplements with a permissible content of 10 mg of MK, the preparation I was closest to meeting the requirements, with an average MK content of 85.17% of the value declared by the manufacturer. The highest MK content was recorded in preparation V and was at 122.52% of the declared value. On the other hand, the lowest content was observed in preparation VI, which amounted to only 5.44% of the declared content. Of the 7 dietary supplements tested, the MK content was lower than the manufacturer claimed in 5 cases and higher in 2 cases.

Among the food supplements with a permitted content of less than 3 mg of MK, preparation VIII came closest to meeting the requirements, with an average MK content of 105.89% of the manufacturer’s declared value. On the other hand, the content of the following two preparations (IX and X) was 89.86% and 88.34%, respectively. It should be noted that, for supplement X, the manufacturer recommends taking 1/4 of a tablet. However, this information is indicated on the packaging separately below the table, where the MK content is misleadingly stated as 2.5 mg. A prohibited dose of around 10 mg could, therefore, easily be taken by an inattentive user.

The results obtained indicate that food supplements on the Polish market do not meet the requirements for the quantitative content of MK. However, it should be noted that the analysis was performed on a single series’ package. This does not mean that all dietary supplements on the market do not meet the requirements for quantitative composition. However, the fact that none of the tested preparations contained the amount of MK declared by the manufacturer raises questions about whether food supplements are adequately controlled.

Gulia Nannoni et al. demonstrated in their study that, for extracts from natural fermented red rice, the ratio of MK-A to the sum of lactone and acidic forms of monacolin, expressed as a percentage, is at least 30%. A too low proportion of MK-A may indicate the addition of synthetic lovastatin, which occurs mainly in the lactone form and distorts the natural proportions [35]. For this reason, the analysed ratio is an important criterion for assessing the authenticity and integrity of the extracts, as well as the quality of the fermentation process. Therefore, it can be assumed that a similar percentage of MK-A in supplements derived from natural RYR also may indicate a probable lack of adulteration and the use of appropriate quality natural starting raw material.

**Figure 3:**
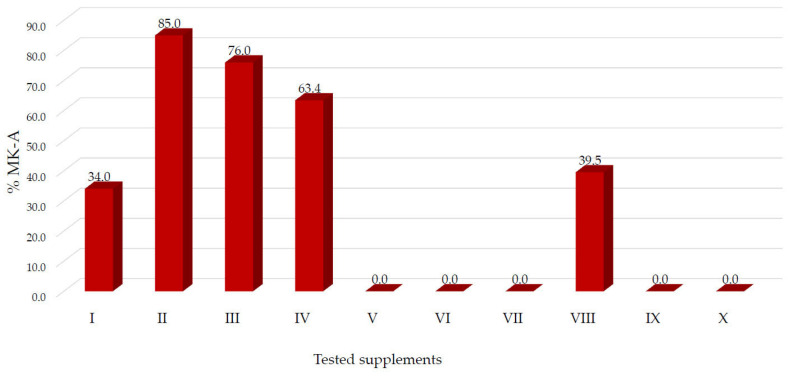
The percentage of MK-A in the tested supplements.

In the supplements tested, the MK-A content ranged from 34.04% to 84.99% for preparations I–IV and VIII. No MK acid form was detected in preparations V–VII and IX–X (Figure 3), which may indicate a compositional profile inconsistent with that expected for non-adulterated RYR-based products.

Analyses of the tested food supplements using the developed method did not detect CTN. Given that the CTN concentration in the supplements may have been below the method’s LOQ and even LOD, an additional LC-MS/MS analyses were performed. These analyses provided unequivocal confirmation that the samples analysed were free of this mycotoxin.

## 4. Discussion

In the scientific literature, MK is frequently classified and described as a nutraceutical with well-documented cholesterol-lowering effects, particularly in the context of RYR-based products [36]. Nutraceuticals are positioned between food and pharmaceuticals and include functional foods and dietary supplements; however, their biological efficacy is weaker than that of medicinal products and typically requires long-term use [37]. Nutraceuticals include, among others, functional foods, dietary supplements, as well as pro- and prebiotics. Their efficacy is considerably weaker than that of medicinal products; therefore, achieving the desired effects requires prolonged use.

The consumption of dietary supplements, including nutraceuticals, is very high and continues to grow, driven by consumers’ belief that these products are natural and therefore free from risks. Unfortunately, the quality and safety of the production of raw materials and finished supplements represent a widespread concern for consumers, healthcare providers, and regulatory authorities [35].

The most commonly observed issues related to supplements include discrepancies between the actual content of ingredients and the manufacturer’s declaration, adulteration (whether unintentional or deliberate), and contamination, for example, with heavy metals or mycotoxins, which may adversely affect consumer health. Adulteration of natural products can occur through the addition of synthetic drugs, providing tangible economic benefits, as the extraction of nutraceuticals from natural sources, such as plants, is often limited, while the preparation and standardisation of extracts is time-consuming and costly [37]. Monacolin K is popular due to its biological properties; however, insufficient quality control of dietary supplements raises concerns regarding the reliability of manufacturers’ claims. Commission Regulation (EU) No 212/2014 indicated that CTN levels in certain RYR products may be sufficiently high to pose a risk of nephrotoxicity [27]. Righetti et al. detected CTN in all analysed RYR supplements, frequently at concentrations exceeding EU limits. Many products also did not meet GMP requirements regarding MK content, and additionally, low-dose simvastatin contamination was detected. These findings highlight significant shortcomings in market surveillance [9]. Further analytical studies using HPLC-DAD revealed anomalies in monacolin profiles, suggesting adulteration of RYR extracts with synthetic lovastatin. Nanoni et al. proposed that one criterion for detecting extract adulteration is the percentage of MK-A relative to the sum of MK-L and MK-A. Both monacolin forms—the acid and lactone—tend to interconvert, with MK-L usually predominating; however, its proportion should not exceed 70% of the combined MK-L and MK-A content. If HPLC indicates a higher MK-L percentage, the product is likely adulterated [35].

In view of the above, the aim of our study was to develop an HPLC-DAD method that could serve not only to verify the declared MK content in supplements but also to determine both of its forms in the presence of other active substances. For the extraction step, methanol was found to be the most suitable solvent for both MK and CTN. In contrast, the extraction efficiency of B vitamins and folic acid increased with increasing solvent polarity, achieved by adding 10 mM phosphate buffer (pH 6) or water to methanol. The results of the optimisation of the extraction mixture were shown in the Supplementary Materials (Figure S3). The potential interconversion between MK-L and MK-A was considered during method development. To minimise this effect, sample storage, extraction, and chromatographic conditions were carefully controlled with respect to solvent composition, pH, temperature, and analysis time. All standards and samples were prepared using the same protocol and analysed immediately after preparation, thereby limiting time-dependent interconversion. Another objective was to assess changes in supplement quality in the context of evolving regulations regarding the permitted MK dose. During method development, we also considered the possibility of CTN determination. The developed method was fully validated and is suitable for routine quality control of supplements containing MK-L and MK-A in the presence of four B-group vitamins and CTN. It ensures high selectivity, sensitivity, and accuracy. To our knowledge, no method has been reported that allows simultaneous determination of MK-L, MK-A, and CTN in the presence of four B-group vitamins and phytosterols in the available literature [20,21,22,23,24,25,26,27,28,29]. The developed HPLC-DAD method employs a C18-PFP column, which offers several significant advantages compared to the C18 column used in analytical methods described in the literature. Firstly, due to the presence of pentafluorophenyl groups, the stationary phase provides enhanced selectivity resulting from additional π–π, dipole–dipole, and hydrogen bonding interactions, enabling efficient separation of the lactone and acid forms of MK as well as CTN in supplements with a complex matrix and composition, including vitamins B_1_, B_6_, B_12_, and folic acid. Such broad applicability is not offered by previously published methods, which typically focus on determining monacolins alone [20,21,22] or exclusively CTN [27,29]. Methods employing UHPLC-DAD-MS or UHPLC-QToF-MS, although characterised by higher sensitivity and identification capability [24,25], require access to costly instrumentation, limiting their use in routine quality control of dietary supplements. In contrast, the described method relies solely on HPLC with DAD detection, available in most analytical laboratories, which constitutes a significant practical advantage. Although systematic studies specifically addressing batch-to-batch reproducibility of PFP stationary phases are scarce, the literature on stationary phase chemistry suggests that phases exhibiting multiple interaction mechanisms may be more sensitive to subtle differences in surface properties than classical C18 phases. This increased sensitivity is commonly attributed to the complexity of PFP surface chemistry and the contribution of multiple retention mechanisms. Consequently, even small differences in ligand density, endcapping, or surface coverage between production batches may lead to measurable changes in retention behaviour and selectivity. Although the C18–PFP column used in this study provided superior resolution for polar compounds compared with a classical C18 phase, the potential impact of inter-batch variability should be considered when transferring the method or applying it in routine analyses.

Among the key benefits of the new method is also the ability to simultaneously determine several chemically distinct groups of compounds within a single chromatographic analysis, which has not been previously reported in the literature dedicated to monacolins and CTN. UHPLC methods, although generally much faster in terms of analysis time (5–21 min) and more sensitive [25,27], are designed for narrow groups of analytes and do not encompass a broad profile of diverse compounds. In this context, the presented method provides a more comprehensive solution, useful for a complete assessment of the quality of RYR-based dietary supplements. On the other hand, the HPLC-DAD method exhibits certain limitations compared to UHPLC-MS/MS or UHPLC-FL, particularly in terms of sensitivity. The limits of detection and quantification for MK-L, MK-A, and CTN (0.96 and 2.85 µg/mL; 0.91 and 2.77 µg/mL; 2.18 and 3.48 µg/mL, respectively) are higher than those achieved with methods employing mass or fluorescence detection [16,27], which reach ng/mL levels or lower. An additional limitation is the extended chromatographic analysis time (33 min), exceeding typical UHPLC times (5–12 min for CTN, 12–21 min for monacolins) [25,29], which also entails greater solvent consumption and lower analytical throughput. Furthermore, the method does not provide structural identification of compounds via mass spectrometry, which is standard for reference methods for determining monacolins and *Monascus* metabolites [24,28]. Despite these limitations, the developed method represents a valuable tool for routine quality control of RYR-based dietary supplements, particularly in settings where access to UHPLC-MS/MS instrumentation is limited. Its unique advantage lies in the ability to simultaneously determine MK-L and MK-A as well as CTN in the presence of B-group vitamins, making it especially useful for quality control of multi-component food supplements.

The analysis of dietary supplements available on the Polish market revealed discrepancies between the manufacturers’ declarations and the actual MK content. It is particularly concerning that, despite the recommendation to reduce the MK dose below 3 mg, a preparation containing 10 mg of MK per tablet is being marketed, with only the information provided on the packaging stating that one-quarter of a tablet should be taken daily. This situation may pose a risk of users taking an unauthorised 10 mg dose by mistake. Furthermore, the evaluated ratio of the acidic form MK-A to the sum of MK-A and MK-L suggested potential adulteration of certain products with synthetic lovastatin, as MK-A was not detected in several supplements. Analyses of the supplements, performed using the developed method and confirmed by LC-MS/MS, demonstrated the absence of CTN.

## 5. Conclusions

The developed and validated RP-HPLC-DAD method provides a robust and efficient approach for the simultaneous determination of MK in acidic and lactone forms as well as CTN in complex formulations containing B vitamins (B_1_, B_6_, B_12_) and folic acid. Its compliance with international guidelines (ICH) confirms its suitability for routine quality control in food supplements. By ensuring accurate quantification of these compounds, the method supports regulatory standards and enhances consumer safety. Studies have indicated that, in the tested samples, the MK content did not always fall within the manufacturer’s declared range. It should be noted that our investigation was limited to a few preparations from a broad market and involved a small sample size restricted to single product batches. Therefore, further testing of MK supplements remains important to support consumer safety, evaluate efficacy, and help establish appropriate quality standards.

## Figures and Tables

**Table 1:** The gradient elution programme of the mobile phase.

Time [min]	Phosphate Buffer [%]	Acetonitrile [%]
0–3	95	5
3–10	95 → 50	5 → 50
10–18	50 → 30	50 → 70
18–23	30 → 30	70 → 70
23–28	30 → 95	70 → 5
28–33	95	5

**Table 2:** Separation parameters for the analysed substances at 230 nm.

Compound	t_R_ (min)	k	N	As (*n* = 5)	Rs ^a^ (*n* = 5)
B_1_	2.80	0.14	7332	1.35	-
B_6_	4.21	0.72	10,805	0.97	5.31
B_12_	10.57	3.31	148,441	1.23	46.74
FA	10.87	3.43	107,256	1.14	2.47
CTN	17.22	6.03	122,769	1.24	2.11
MK-A	19.00	6.75	180,425	1.13	9.48
MK-L	21.59	7.81	163,766	1.22	2.56

^a^ Resolutions were calculated between two adjacent peaks. t_R_—retention time; k—retention coefficient; Rs—resolution; N—number of theoretical plates; As—asymmetry factor.

**Table 3:** Summary of linearity parameters and detection limits (LOD) and quantification limits (LOQ) for study substances.

Substance	LOD [µg∙mL^−1^]	LOQ [µg∙mL^−1^]	Linearity Range [µg∙mL^−1^]	Regression Parameters			Normality of Residuals (S-W Test) ^b^	
				Coefficients P = ac + b ± S_e_ ^a^	Standard Error	R	p	W
**MK-L**	0.94	2.85	12.48–37.44	a = 1166 × 10^2^ b = −22,500 ± 33,291.0	S_a_ = 1193 S_b_ = 31,582	0.9996	0.1623	0.8883
**MK-A**	0.91	2.77	12.48–37.44	a = 1148 × 10^2^ b = 66,634 ± 31,846	S_a_ = 1141 S_b_ = 30,212	0.9996	0.9584	0.9788
**CTN**	2.18	3.48	3.48–5.22	a = 1055 × 10^2^ b = −1508.0 ± 9917.1	S_a_ = 5098 S_b_ = 22,406	0.9908	0.6606	0.9493

^a^ P = peak area; c = concentration; a and b = regression coefficients, S_e_ = standard error of the estimate, S_a_ = standard deviation of the regression coefficient a; S_b_ = standard deviation of the regression coefficient b; ^b^ normal distribution of residuals if *p* > 0.05; S-W Test—Shapiro-Wilk Test.

**Table 4:** Validation results for precision, indirect precision and recovery of the method.

Substance	Precision, *n* = 6, RSD%	Indirect Precision *n* = 6, RSD%	Recovery, *n* = 3 [%]
**MK-L**	0.47	1.33	x_m_ = 98.73% RSD = 1.23%
**MK-A**	0.70	0.85	x_m_ = 100.64% RSD = 0.78%
**CTN**	0.61	0.91	x_m_ = 99.50% RSD = 3.01%

x_m_—arithmetic mean; RSD = relative standard deviation.

**Table 5:** Results of MK determination in food supplements with statistical evaluation.

Preparation	Declared Content of MK	Determined Content of MK-L (mg)	Determined Content of MK-A (mg)	The Sum of the Determined Content of MK-L and MK-A (mg)	Ratio of Determined to Declared MK Content (%)
**I**	10 mg/tablet	x_m_ = 5.62 RSD = 6.53%	x_m_ = 2.90 RSD = 7.32%	x_m_ = 8.52 RSD = 6.66%	85.17
**II**	10 mg/tablet	x_m_ = 1.13 RSD = 7.27%	x_m_ = 6.40 RSD = 8.11%	x_m_ = 7.53 RSD = 7.73%	75.29
**III**	10 mg/tablet	x_m_ = 1.67 RSD = 4.10%	x_m_ = 5.29 RSD = 3.96%	x_m_ = 6.96 RSD = 3.08%	69.64
**IV**	10 mg/tablet	x_m_ = 2.97 RSD = 4.63%	x_m_ = 5.17 RSD = 4.74%	x_m_ = 8.15 RSD = 4.66%	81.45
**V**	10 mg/capsule	x_m_ = 12.25 RSD = 7.80%	**-**	x_m_ = 12.25 RSD = 7.80%	122.52
**VI**	10 mg/tablet	x_m_ = 0.54 RSD = 5.60%	**-**	x_m_ = 0.54 RSD = 5.60%	5.44
**VII**	10 mg/capsule	x_m_ = 11.56 RSD = 4.33%	**-**	x_m_ = 11.56 RSD = 4.33%	115.89
**VIII**	2.75 mg/capsule	x_m_ = 1.76 RSD = 3.68%	x_m_ = 1.15 RSD = 2.90%	x_m_ = 2.91 RSD = 3.30%	105.89
**IX**	2.90 mg/capsule	x_m_ = 2.61 RSD = 1.75%	**-**	x_m_ = 2.61 RSD = 1.75%	89.86
**X**	10 mg/tablet	x_m_ = 8.83 RSD = 1.61%	**-**	x_m_ = 8.83 RSD = 1.61%	88.34

x_m_—arithmetic mean; RSD = relative standard deviation.

## Data Availability

The data are available from the authors upon reasonable request.

## Supplementary Materials

The following supporting information can be downloaded at: https://www.mdpi.com/article/10.3390/molecules31010016/s1, Figure S1: Chromatogram obtained after the MK-L hydrolysis process as described by Yang and Hwang [33].; Figure S2: Chromatogram obtained after methanol extraction of selected phytosterols (beta-sitosterol, campesterol, stigmasterol); Figure S3: The comparison of extraction methods: I: mixture of 10 mM PBS pH = 6.01:methanol (80:20 *v*/*v*); II: mixture of 10 mM PBS pH = 6.01:methanol (50:50 *v*/*v*); III: methanol:water (90:10 *v*/*v*); IV: 100% methanol. Table S1: System suitability conditions for target analytes.
