# Supplementary material for: Determination of Monacolin K and Citrinin in the Presence of Other Active Ingredients Found in Selected Food Supplements by HPLC-DAD
Source: Molecules. 2025 Dec 20;31(1):16. doi: 10.3390/molecules31010016 (PMC12786744; doi:10.3390/molecules31010016)
Supplement: Supplementary file 1 [file molecules-31-00016-s001.zip › molecules-4043827-supplementary.pdf]

Supplementary materials

Determination of Monacolin K and Citrinin in the Presence of Other Active Ingredients Found in Selected Food Supplements by HPLC-DAD

Urszula Hubicka, Barbara Żuromska-Witek, Marek Szlósarczyk, Ewelina Sołtys, Martyna Rusak and Izabela Gacal

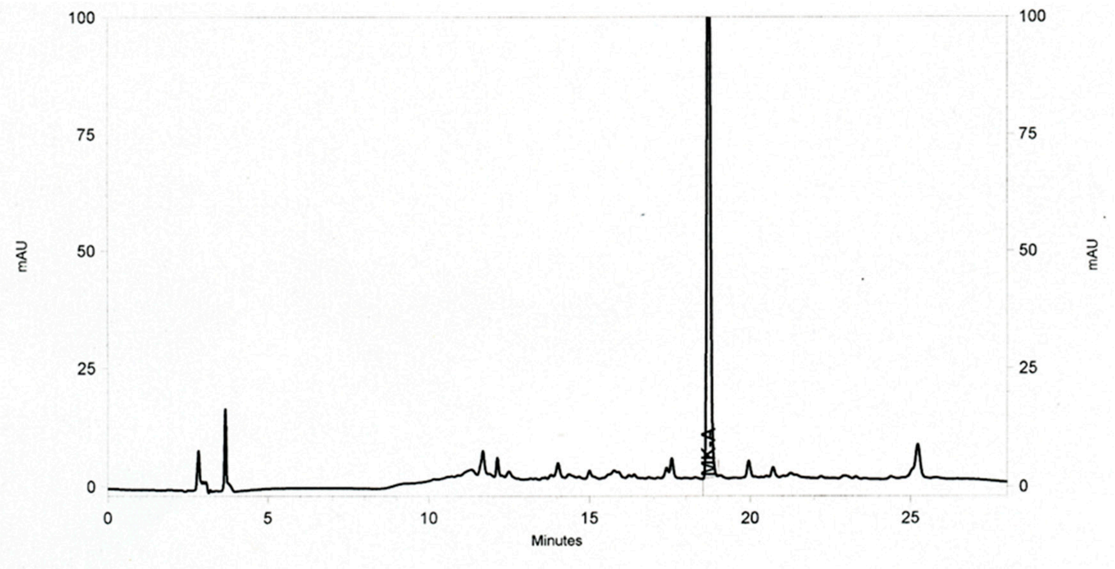

Figure S1. Chromatogram obtained after the MK-L hydrolysis process as described by Yang and Hwang [33].

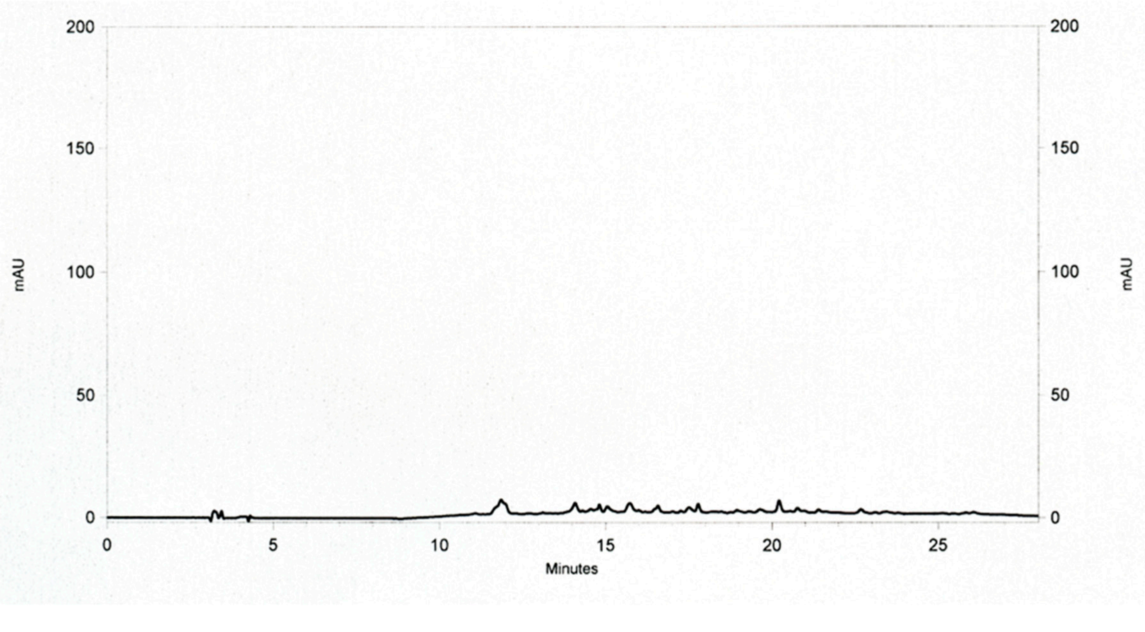

Figure S2. Chromatogram obtained after methanol extraction of selected phytosterols (beta-sitosterol, campesterol, stigmasterol).

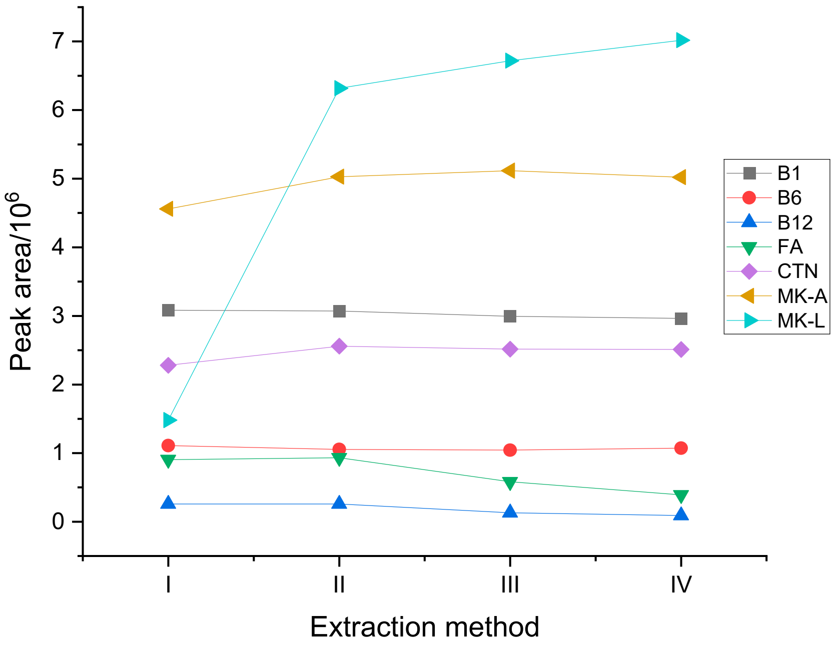

Figure S3. The comparison of extraction methods: I: mixture of 10 mM PBS pH = 6.01:methanol (80:20 v/v); II: mixture of 10 mM PBS pH = 6.01:methanol (50:50 v/v); III: methanol:water (90:10 v/v); IV: 100% methanol.

Table S1. System suitability conditions for target analytes.

MK-A	Retention time (t_R)	Theoretical plates (N)	Asymmetry (As)	Peak area
1	18.65	139,638	1.16	1,791,781
2	18.63	139,801	1.16	1,764,411
3	18.67	139,025	1.15	1,830,431
4	18.61	139,549	1.16	1,831,956
5	18.63	142,885	1.11	1,816,813
Statistical analysis	$\bar{x}$=18.64<br>SD=0.02<br>RSD=0.12%	$\bar{x}$=140179.6<br>SD=1540.02<br>RSD=1.10%	$\bar{x}$=1.15<br>SD = 0.02<br>RSD=1.88%	$\bar{x}$=1,807,078<br>SD = 28781.6<br>RSD=1.59%
**MK-L**	**Retention time (t_R)**	**Theoretical plates (N)**	**Asymmetry (As)**	**Peak area**
1	21.19	167,069	1.11	3,736,053
2	21.18	163,125	1.11	3,752,852
3	21.21	165,469	1.14	3,793,801
4	21.16	163,718	1.14	3,815,526
5	21.17	165,756	1.14	3,616,529
Statistical analysis	$\bar{x}$= 21.18<br>SD=0.02<br>RSD=0.09%	$\bar{x}$=140,179.6<br>SD=1599.0<br>RSD=0.97%	$\bar{x}$=1.13<br>SD = 0.02<br>RSD=1.45%	$\bar{x}$=3,742,952<br>SD = 77,429.0<br>RSD=1.59%

CTN*	**Retention time (t_R)**	**Theoretical plates (N)**	**Asymmetry (As)**	**Peak area**
1	16.85	123,584	1.09	449,724
2	16.9	124,780	1.15	454,,118
3	16.92	124,089	1.12	442122
4	16.9	124,773	1.14	449,139
5	16.92	125,379	1.12	458,260
Statistical analysis	$\bar{x}$= 16.90<br>SD=0.03<br>RSD=0.17%	$\bar{x}$=1,240,521.0<br>SD=694.86<br>RSD=0.56%	$\bar{x}$=1.12<br>SD = 0.02<br>RSD=2.04%	$\bar{x}$=450,672.6<br>SD = 6037.5<br>RSD=1.34%

*The citrinin was detected at different 325 nm wavelength.
